# High-Temperature-Resistant Profile Control System Formed by Hydrolyzed Polyacrylamide and Water-Soluble Phenol-Formaldehyde Resin

**DOI:** 10.3390/gels10060413

**Published:** 2024-06-20

**Authors:** Xuanran Li, Shanglin Liu, Juan Zhang, Shujun Han, Lun Zhao, Anzhu Xu, Jincai Wang, Fujian Zhou, Minghui Li

**Affiliations:** 1 Research Institute of Petroleum Exploration & Development, Beijing 100083, China; 2 National Key Laboratory of Petroleum Resources and Engineering, China University of Petroleum (BeiJing), Beijing 102249, China; 3 Unconventional Petroleum Research Institute, China University of Petroleum (Beijing), Beijing 102249, China

**Keywords:** heavy oil reservoir, profile control, hydrolyzed polyacrylamide, phenol-formaldehyde resin, gel

## Abstract

To realize the effective profile control of a heavy oil reservoir, hydrolyzed polyacrylamide (HPAM) and water-soluble phenol-formaldehyde resin (PR) were chosen to prepare the profile control system, which gelled at medium or low temperatures and existed stably at high temperatures in the meantime. The effects of phenolic ratios, PR concentration, and HPAM concentration on the formation and strength of the gels were systematically studied by the gel-strength code method and rheological measurements. And the microstructure of the gels was investigated by scanning electron microscope measurements. The results showed that the gelling time of the HPAM-PR system was 13 h at 70 °C. The formed gel could stay stable for 90 days at 140 °C. In addition, the gels showed viscoelastic properties, and the viscosity reached 18,000 mPa·s under a 1.5 s^−1^ shearing rate due to their three-dimensional cellular network structure. The formation of the gels was attributable to the hydroxyl groups of the PR crosslinking agent, which could undergo the dehydration condensation reaction with amide groups under non-acidic conditions and form intermolecular crosslinking with HPAM molecules. And the organic crosslinker gel system could maintain stability at higher temperatures because covalent bonds formed between molecules.

## 1. Introduction

In recent years, the steam-driven recovery of thick oil reservoirs has received increasing attention. In order to improve the non-homogeneous formation environment, some technologies have been performed, such as acidizing, profile control technology et al. [1,2]. Deep profile control technology is achieved by injecting a conditioning agent to seal the hypertonic layer. There are many types of plugging agents, among which gel-type plugging agents are most widely used in practical applications [3]. The gel-plugging agent is usually made of the polymer as the main agent, a crosslinking agent, stabilizer, additives, etc., which is relatively inexpensive, stratigraphically selective, and has a good plugging effect. During the injection process, the crosslinking solution is prioritized to enter the high-permeability layer to form gel blocking. During the migration of the gel in the pore throat, the gel is stretched due to the space limitation of the pore throat. Meanwhile, the gel can change shape to pass through the pore throat; thereby, it has a strong flow capacity. And the strength of the gel can prevent the occurrence of the scuttling phenomenon so that the steam enters the medium and low permeability reservoirs and improves the recovery rate of the reservoir [4]. High-temperature steam experiences heat loss during the injection process and becomes hot water or steam at 140 °C~190 °C after reaching the target formation. In order to adapt to the high-temperature environment after steam throughput, the gel-plugging agent should meet the following two requirements: firstly, a good gel-forming performance in low and medium-temperature formations to meet the plugging of high-permeability formations; secondly, the performance of the gel should remain stable in the high-temperature steam environment [5,6].

At present, the gel-plugging technology applicable to medium- and low-temperature reservoirs with formation temperature no higher than 90 °C is relatively mature, and the most widely used gel-plugging agents mainly include inorganic plugging gels, PAM/metal ion crosslinking gels, PAM/organic crosslinking agent gels, etc. However, these gel-type plugging systems show clear shortcomings in their comprehensive performance. On the one hand, the gel is good, but the maximum temperature resistance does not exceed 130 °C; on the other hand, the temperature performance is good, but the gel is too fast (<30 min) and cannot realize the effective injection of the scene [7]. For high-temperature reservoirs with temperatures higher than 90 °C, in order to improve the non-homogeneous environment of the formation and enhance the oil recovery effect, the research and development of the high-temperature profiling agent has become an important topic. In order to ensure the sealing effect on the high permeability layer, such regulators are required to have good temperature resistance [8]. However, the currently reported high-temperature-resistant regulator system has too high gel-forming temperatures due to the organic crosslinking agent and cannot be gel-forming at low temperatures [9]. Therefore, there is an urgent need to develop a gel-plugging agent with high gel formation strength at low and medium temperatures and good temperature resistance at high temperatures in order to satisfy the demand for the steam throughput of thick oil reservoirs and to improve crude oil recovery [10].

The commonly used gel crosslinking systems mainly include the HPAM/inorganic metal ion gel system and phenolic resin organic crosslinking system [11]. The HPAM/inorganic metal ion gel system is usually used as an anatomical water-plugging agent or fracturing fluid in oilfield production, and the reaction mechanism is that carboxylate and high-valent metal ions in the polyacrylamide undergo a crosslinking reaction through ligand bonds [12,13,14,15], among which the most reported related studies use a chromium acetate crosslinker. DiGiacomo [16] characterized polyacrylamide and trivalent chromium ion gels by a ^13^C nuclear magnetic resonance based on the paramagnetism of Cr^3+^ and found that chromium ions in the complexed or free state crosslinked with carboxylates on HPAM molecules through ligand bonding to form a three-dimensional reticulated gel skeleton, yielding a strong gel with good viscoelasticity. The chromium ion crosslinking agent is usually used in reservoirs with formation temperatures below 70 °C. Too-high gel formation temperatures will lead to a violent reaction, and it is difficult to control its gel formation time and strength. Due to the fast reaction rate of Cr^3+^ and the polymer, the crosslinking time can be prolonged by introducing divalent chromium ions together with the use of reducing agents to reduce hexavalent chromium ions to trivalent chromium ions in the formation or injection process in the related research [17]. However, due to the carcinogenic properties of divalent chromium ions, the complexes of Cr^3+^ (chromium acetate [18,19], chromium malonate, etc.) are usually used as a substitute, which can also effectively delay the gel formation time. The ligand bonds formed by COO- and Cr^3+^ in chromium gels are easy to break at high temperatures above 100 °C. Compared with metal ion crosslinker gel systems, organic crosslinker gel systems are able to remain stable at higher temperatures through the formation of covalent bonds between molecules [20]. One of the most widely used crosslinkers is phenolic resin crosslinkers and commonly used phenols include phenol, hydroquinone, and resorcinol, etc., and commonly used aldehydes include formaldehyde and hexamethylene tetramine (urotropin). The phenolic resin gel conditioning system has good temperature resistance regarding the gel formed at high temperatures, but its gel formation is slow and weak at low temperatures, which is due to the high activation energy of the organic reactive groups and the lower gel formation temperature makes the crosslinking reaction proceed slowly [21,22].

Therefore, in this paper, starting from the synthesis method of phenolic resins, a resin crosslinker with higher activity was synthesized by adjusting the phenolic-to-formaldehyde ratio, which, in turn, improved its gel formation rate at low temperatures to prepare a high-strength temperature-resistant gel system. A temperature-resistant HPAM was selected, and the properties of HPAM and different phenolic resin crosslinker systems in terms of gel-forming strength, gel-forming time, dehydration rate, and stability at different temperatures were investigated to prepare a PR crosslinking system that was low-cost, highly stable, and suitable for steam drive in medium-to low-temperature reservoirs.

## 2. Results and Discussion

### 2.1. Effect of Phenolic Ratio of the PR Crosslinkers on the Gel Formation and Strength

The gelation of the 0.8 wt% PR crosslinker and HPAM with different concentrations at 50 °C and the stability of the obtained gels at 140 °C are shown in Table 1. It was seen that the bigger the *r*_F/P_ value, the stronger the gel strength. When *r*_F/P_ was one, the maximum gel strength was E, while when *r*_F/P_ was three, the maximum gel strength was G at 50 °C. This was because, with the increase in the formaldehyde amount, the same volume of resin contained more hydroxymethyl groups, which had high chemical activity. Under alkaline conditions, these hydroxymethyl groups could react with the amide groups on the polyacrylamide molecular chain, resulting in crosslinking between molecules, thereby improving the reaction rate and enhancing the strength of gel formation. The gels with high strength (≥F) were stored at 140 °C to evaluate their high-temperature-resistance performance. Dehydration occurred for the gels with 0.6 wt% HPAM. The strength of the gels with 0.8 wt% HPAM and *r*_F/P_ ≤ 3 was D grade after 30 days. It was worth noting that the strength of the gels with *r*_F/P_ = 4 reached E grade even after 90 days. Moreover, the *r*_F/P_ had a great effect on the gelation time. In particular, when the HPAM concentration was 0.6 wt%, with the increase in *r*_F/P_, the gelation time decreased from 160 h to 31 h. It could be seen that the overall reaction time of the PR-HPAM gel was still slow at the reaction temperature of 50 °C. Increasing the *r*_F/P_ to improve the reactivity of oligomers of resin crosslinkers was an effective method to accelerate the reaction rate and increase the gel strength at medium and low temperatures.

**Figure 1:**
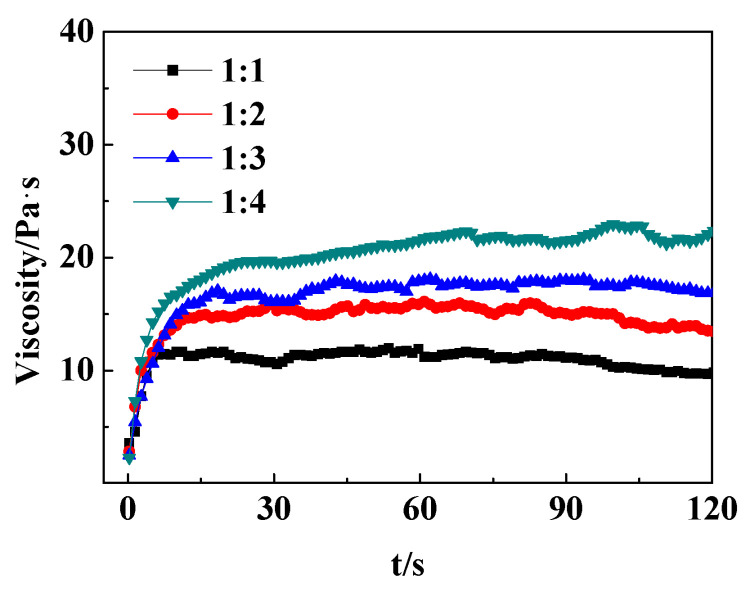
Effect of different phenolic ratios on gel viscosity (0.8 wt% HPAM + 0.8 wt% PR, T = 90 °C).

The gelation of the 0.8 wt% PR crosslinker and HPAM with different concentrations at 90 °C and the stability of the obtained gels at 140 °C are shown in Table 2. The gelation temperature also had clear effects on the gelation time, gel strength, and high-temperature resistance performance. The influence of the *r*_F/P_ on the profile control system at 90 °C was clearer than that at 50 °C, which reflected the higher gel strength, faster gelation time, and longer temperature resistance at 140 °C. This was because in the high-temperature environment, the activation energy of the amide group of polyacrylamides and the hydroxymethyl in the resin was significantly increased, and the intermolecular movement was more intense, which promoted the crosslinking and entanglement of the thermoplastic skeleton of the water-soluble resin and the polyacrylamide skeleton, and thus improved the overall stability of the gel. It could be seen that when the HPAM concentration was 0.8 wt%, with *r*_F/P_ increasing, the gel strength increased from E to G grade, and the shear viscosity increased from 10,000 mPa∙s to 19,000 mPa∙s, as shown in Figure 1. In addition, when the HPAM concentration was higher than 0.8 wt%, the gel with *r*_F/P_ < 2 showed poor temperature resistance, while the gel strength was F when *r*_F/P_ = 4. Therefore, the ideal *r*_F/P_ was four in order to obtain the profile gel system with high gel strength and good temperature resistance performance.

### 2.2. Effect of HPAM Concentration on the Gel Formation and Strength

**Figure 2:**
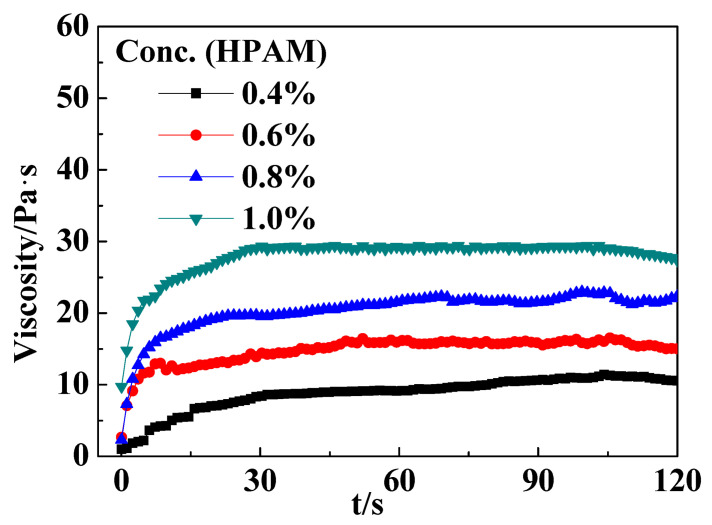
Effect of different polymer concentrations on gel viscosity (T = 90 °C; γ = 1 s^−1^).

The gelation of 0.8 wt% PR with *r*_F/P_ = 4 and HPAM with different concentrations at 90 °C and the stability of the obtained gels at 140 °C are shown in Table 3. The results show that the gel strength increased with the increase in the HPAM concentration. When the HPAM concentration was ≥0.6 wt%, the gel strength was G or H grad. However, the gel with 0.6 wt% HPAM became severely dehydrated at 140 °C. The gels with 0.6 and 0.8 wt% HPAM showed good temperature resistance and remained stable after 90 days. Figure 2 presents the shearing viscosities of the gels with 0.8 and 1.0 wt% HPAM at about 20 Pa∙s and 30 Pa∙s, respectively, which meets the viscosity and strength requirements of the profile control system.

### 2.3. Effect of PR Crosslinker Concentration on the Gel Formation and Strength

In total, 0.8 wt% of the HPAM solution and the 0.3 wt%–1.0 wt% PR crosslinker (*r*_F/P_ = 4) were mixed together to gel at 50 °C, 70 °C, and 90 °C, and the gelation state was observed. Gels with a strength greater than F were placed in a constant temperature oven at 140 °C to assess their thermal stability. The results are shown in Table 4. It was observed that the greater the content of the PR crosslinker, the higher the gel strength. When the PR resin mass fraction was 0.3 wt%, the gelation rate of the mixture at 50 °C and 70 °C was slow, indicating that the low concentration of the crosslinker and the low crosslink density with the polymer backbone resulted in weak gel strength. When the PR resin mass fraction was ≥0.8 wt%, i.e., when the mass ratio of PR resin to polymer was one, the gel strength reached the G level. Therefore, the PR crosslinker mass fraction needed to be at least 0.8 wt%. The thermal stability test results show that when the PR crosslinker mass fraction was 0.8 wt%, the gel exhibited good thermal stability and retained E-level strength after 90 days. When the PR crosslinker mass fraction was 1.0 wt%, the gel was dehydrated at high temperatures, and the gel volume shrank significantly. Considering all factors, the optimal concentration of the PR crosslinker was determined to be 0.8 wt%, at which point the mass ratio of HPAM to crosslinker was exactly one.

According to the above experimental results, when the resin crosslinking agent content was low, the crosslinking with the active sites on the polymer was incomplete, resulting in an unstable gel structure. Conversely, when the crosslinking agent content was too high, the actual number of crosslinking points involved in the reaction exceeded the number required for forming a stable three-dimensional structure, leading to a deterioration in the stability of the gel structure.

### 2.4. Rheological Properties of the Gels

**Figure 3:**
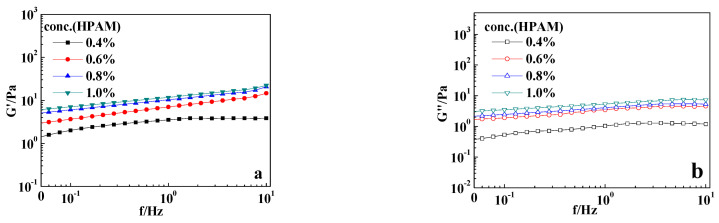
Oscillatory rheological results of the typical samples with different HPAM concentrations: (**a**) the elastic modulus G′; (**b**) the viscous modulus G″. The PR resin (*r*_F/P_ = 4:1) concentration was 0.08 wt%. T = 90 °C.

A crosslinking system was prepared with 0.4 wt%–1.0 wt% HPAM and 0.08 wt% PR resin (*r*_F/P_ = 4:1). The system was placed in an oven at 70 °C for gelation over 3 days. The viscoelasticity of the formed gel was measured using a HAAKE rheometer, and the test results are shown in Figure 3. From Figure 3, it can be seen that in the frequency sweep range of 0–10 Hz, the elastic modulus G′ and the viscous modulus G″ values of the PR gels showed typical properties of gels. Thhe elastic modulus was higher than the viscous modulus within the measured range. As the HPAM concentration increased from 0.4 wt% to 1.0 wt%, the G′ values of the gels initially increased uniformly and then increased slowly, indicating that the gel strength was significantly enhanced by a moderate increase in polymer concentration. When the HPAM concentration reached 0.8 wt%, the gel strength began to increase slowly, suggesting that the gel became well crosslinked at this concentration. The viscous modulus G″ of the gel showed a trend of first increasing and then decreasing with the increase in shear frequency, likely due to the shear thinning of the gel under high-frequency shear [23]. As the polymer concentration increased, the G″ values of the gel increased uniformly, indicating that the increase in polymer concentration promoted higher viscosity. This was consistent with the results observed using the visual inspection method.

**Figure 4:**
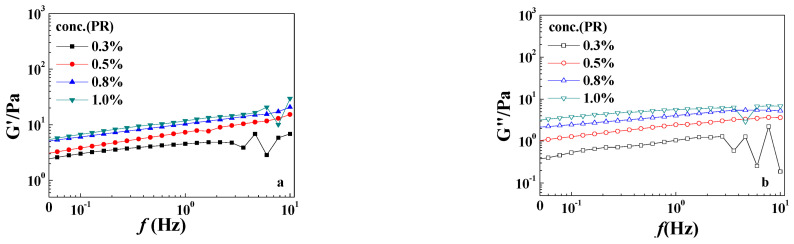
Oscillatory rheological results of the typical samples with different PR concentrations (*r*_F/P_ = 4): (**a**) the elastic modulus G′; (**b**) the viscous modulus G″. The HPAM concentration was 0.8 wt%.

The effects of PR crosslinker concentration on the rheological properties of the gels are shown in Figure 4. With the increase in the PR crosslinker concentration, both G′ and G″ increased. At the same time, both the elastic modulus and the viscous modulus increased with the increase in frequency, and the amplitude of the increase remained the same, which is a typical rheological property of the hydrogel phase.

### 2.5. Microstructure of the Gels

**Figure 5:**
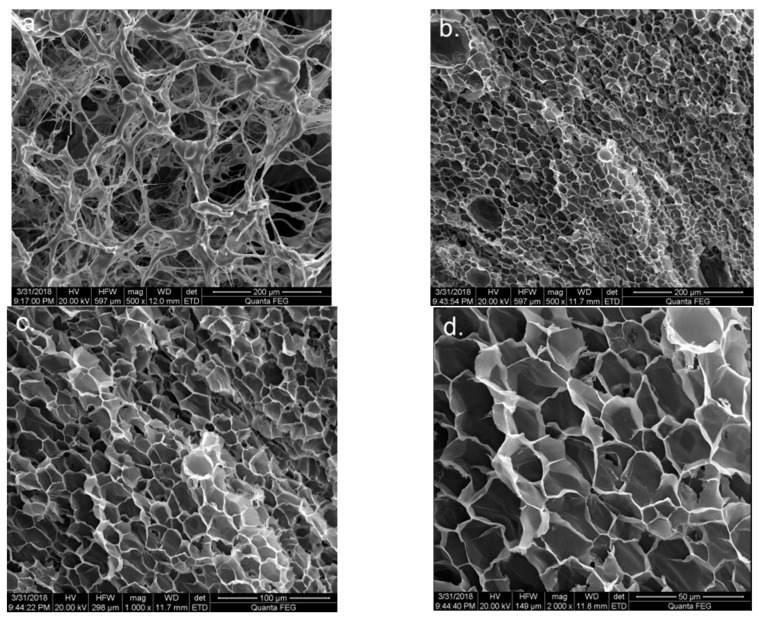
The microstructures of the gel system with 0.8 wt% HPAM and 0.8 wt% PR (*r*_F/P_ = 4): (**a**) the SEM image of the solution before gelation; (**b**–**d**) the SEM images of the solution after gelation with different magnification.

The microstructures of the sample with 0.8 wt% HPAM and 0.8 wt% PR (*r*_F/P_ = 4) before and after gelation at 90 °C are shown in Figure 5. Figure 5a shows that the solution before gelation displayed a chaotic branch-shaped mesh structure with sparse skeletons and large pores. However, after gelation, as shown in Figure 5b, the gel represented a high-density three-dimensional mesh structure similar to a honeycomb, which was attributed to its high viscoelasticity and high-temperature stability. Furthermore, it can be seen from Figure 5c,d that the gel had tight connections between the pores, a higher density of three-dimensional pores, and pore sizes ranging from 20 to 40 µm, which also made the gel more stable at high temperatures.

Based on the above results, it was speculated that the formation of the gels was attributable to the hydroxyl groups of the PR crosslinking agent, which could undergo a dehydration condensation reaction with amide groups under non-acidic conditions and undergo intermolecular crosslinking with HPAM molecules [20]. And the organic crosslinker gel system could maintain stability at higher temperatures because covalent bonds formed between the molecules.

## 3. Conclusions

The phenolic ratios, PR concentration, and HPAM concentration had great effects on the formation and strength of the gel profile control system. Specifically, with the increase in the phenolic ratio, PR concentration, and HPAM concentration, the gel strength was significantly enhanced, and a longer temperature resistance time was obtained. The preferred formulation of the gel system was 0.8 wt% HPAM and 0.8 wt% of the PR crosslinker, which could stay stable for 90 days at 140 °C. In addition, the viscosity of the gel system reached 18,000 mPa·s under a 1.5 s^−1^ shearing rate due to its three-dimensional cellular network structure with a pore diameter of 20 μm.

## 4. Materials and Methods

### 4.1. Materials

Phenol (purity > 99%) was purchased from Beijing Fuchen Chemicals Co., Ltd. (Beijing, China). Formaldehyde (37% solution in water) and calcium silicate (purity > 99%) were obtained from Beijing Yili Fine Chemicals Co., Ltd. (Beijing, China) Sodium hydroxide (purity > 99%) was bought from Beijing Modern Oriental Fine Chemicals Co., Ltd. (Beijing, China) HPAM with a molecular weight of 5–7 million and hydrolysis degree of 25% was obtained from Aisen Flocculant (Taizhou, China) Co., Ltd. Deionized water was made in a laboratory.

Different types of water-soluble PR crosslinkers were synthesized by adjusting the ratio of formaldehyde and phenol (*r*_F/P_) to 1:1, 1:2, 1:3, and 1:4, respectively. Firstly, the melted phenol was placed in a three-necked, round-bottomed flask with the water running through the condensing pipe. Then, the reaction catalyst NaOH solution was dropped into the flask and reacted with phenol for 20 min under stirring conditions. Nitrogen was continuously injected into the reaction system at a certain rate during the reaction process. After the NaOH drops were added, the temperature of the water bath was raised to 75.5 °C. Next, a certain amount of formaldehyde solution was dropped into the flask with a constant pressure drip funnel, and the reaction was carried out for 3 h to prepare the base-catalyzed phenolic resin. The obtained product was stored at 5 °C.

### 4.2. Preparation of the Gels

A certain mass of HPAM was added into deionized water, which was then stirred continuously for 4 h at the stirring rate of 400 r/min until the solution was homogeneous and transparent. Then, the solution was stored for 12 h to make it fully hydrolyze. Next, a certain amount of PR crosslinkers and heat stabilizer was added to the polymer solution, which was stirred at 400 r/min. The mixed solution was stirred for 2 h and then injected into ampoules, which were vacuumed and sealed. All the ampoules were then stored under a set temperature to observe the gelation of the solutions.

### 4.3. The Gel-Strength Code

The strength of the gels was evaluated by the gel-strength code reported by Sydansk [24], as shown in Table 5. When using the gel-strength code method, the same volume of the samples should be placed in the same container for observation. Specifically, the gel strength was evaluated visually by reversing the ampoule containing the gel at 180° and observing the flow state of the gel inside the bottle.

### 4.4. Evaluation Method of Gel Formation Time

The ampoule containing the solution was put in a constant temperature oven to observe the gel formation process every hour. The gel formation time was identified when the ampoule was tilted at 45°, and the liquid surface appeared uneven. The final gelation time was identified when the ampoule was placed horizontally for 30 s, and the gel did not flow.

### 4.5. Evaluation Method of High-Temperature Stability of Gels

The high-temperature stability of the gel was evaluated by the visual inspection method. The ampoule that had been gelatinized at a low temperature was placed in a high-temperature oven at 140 °C. The ampoule was turned over at certain intervals to record the gel flow, degradation and dehydration, and the change in gel strength was determined by the strength code method; the high-temperature stability of the gel was also evaluated by observing the gel strength, and dehydration rate.

### 4.6. Rheological Properties of Gels Measurements

The experiment was carried out by the RS600 rheometer (Thermo Fisher Scientific, Waltham, MA, USA), the cone-plate-measuring rotor system C60/1°Ti was selected, and the experimental measurements were all carried out at 25 °C. The test sampling process should avoid the use of syringes and other apparatus that would produce pre-shear interference with the sample. Therefore, a small spoon was used to take a spoonful of the sample placed in the center of the sample stage. Conditions for the determination of the viscosity of the gel conditioner are as follows: temperature T = 25 °C and shear rate γ = 1.5 s^−1^ under the continuous measurement of 120 s to obtain the viscosity curve of the gel, which tends to stabilize the viscosity of the gel. The conditions for the determination of the viscoelasticity of the gel conditioner are as follows: the viscoelastic curve of the gel is obtained after measuring the frequency *f* = 0.1–10 Hz under the temperature T = 25 °C and stress τ = 1 Pa, which is the viscoelastic modulus curve of the gel.

### 4.7. Scanning Electron Microscope (SEM) Measurements

A Quanta 200F (FEI Company, Hillsboro, OR, USA) scanning electron microscope was used for observation, with the accelerating voltage set to 20 kV and the scanning mode in a high vacuum mode. The sample was first freeze-dried, and then a sample sheet of approximately 4 mm^2^ was taken and affixed to the sample plate using conductive tape in sequence. The sample was frozen with liquid nitrogen and then quickly freeze-dried in a freeze-dryer, sublimating the water to create a dry sample. Gold spray coating was applied to render the sample conductive and prevent charge accumulation, as well as to mitigate thermal damage to the sample. The sample plate was placed within the SEM sample chamber to obtain the photographs.

## Figures and Tables

**Table 1:** Optimizing phenolic ratios for the PR gel system (T = 50 °C).

HPAM Conc.	*r*_F/P_	Gelation Time/h	140 °C			
			0 Day	30 Days	60 Days	90 Days
0.4%	1:1	186	C	-	-	-
	1:2	132	C	-	-	-
	1:3	75	D	-	-	-
	1:4	38	D	-	-	-
0.6%	1:1	160	E	-	-	-
	1:2	118	E	-	-	-
	1:3	63	F	Dehydration	Dehydration	Dehydration
	1:4	31	F	Dehydration	Dehydration	Dehydration
0.8%	1:1	124	E	-	-	-
	1:2	90	F	F	C	-
	1:3	47	G	F	D	D
	1:4	24	G	F	F	E
1.0%	1:1	102	E	-	-	-
	1:2	72	F	F	C	-
	1:3	36	G	F	E	D
	1:4	16	G	F	F	E

**Table 2:** Optimizing phenolic ratios for the PR gel system (T = 90 °C).

HPAM Conc.	*r*_F/P_	Gelation Time/h	140 °C			
			0 Day	30 Days	60 Days	90 Days
0.4%	1:1	15	D	-	-	-
	1:2	13	D	-	-	-
	1:3	10	E	-	-	-
	1:4	8	E	-	-	-
0.6%	1:1	12	E	-	-	-
	1:2	10	F	D	B	-
	1:3	8	F	E	E	C
	1:4	5	G	Dehydration	Dehydration	Dehydration
0.8%	1:1	10	E	-	-	-
	1:2	8	F	F	C	-
	1:3	6	G	F	E	C
	1:4	4	G	G	F	F
1.0%	1:1	8	F	E	C	-
	1:2	6	G	F	C	-
	1:3	5	G	F	F	C
	1:4	3	H	G	F	F

**Table 3:** Optimizing polymer concentration for PR gel system (T = 90 °C).

*r*_F/P_	HPAM Conc.	Gelation Time/h	140 °C			
			0 Day	30 Days	60 Days	90 Days
1:4	0.4%	8	E	-	-	-
	0.6%	5	G	Dehydration	Dehydration	Dehydration
	0.8%	4	G	G	F	F
	1.0%	3	H	G	F	F

**Table 4:** Optimizing crosslinker PR concentration for the PR gel system.

Temperature	PR Conc.	Gelation Time/h	140 °C			
			0 Day	30 Days	60 Days	90 Days
50 °C	0.3%	-	C	-	-	-
	0.5%	39	E	-	-	-
	0.8%	24	G	F	F	E
	1.0%	20	G	Dehydration	F	E
70 °C	0.3%	-	D	-	-	-
	0.5%	20	E	-	-	-
	0.8%	13	G	G	F	E
	1.0%	10	G	Dehydration	Dehydration	Dehydration
90 °C	0.3%	7	E	-	-	-
	0.5%	6	F	E	B	B
	0.8%	4	G	G	F	E
	1.0%	3	H	Dehydration	Dehydration	Dehydration

**Table 5:** The gel-strength code [24].

Code	Gel Types	Gel Strength Description
**A**	No detectable gel formed	The gel appears to have the same viscosity (fluidity) as the original polymer solution, and no gel is visually detectable.
**B**	Highly flowing gel	The gel appears to be only slightly more viscous (less fluid) than the initial polymer solution.
**C**	Flowing gel	Most of the clearly detectable gel flows to the bottle cap upon inversion.
**D**	Moderately flowing gel	Only a small portion (about 5 to 15%) of the gel does not readily flow to the bottle cap upon inversion, which is usually characterized as a “tonguing” gel.
**E**	Barely flowing gel	The gel can barely flow to the bottle cap and/or a significant portion (>15%) of the gel does not flow upon inversion.
**F**	Highly deformable nonflowing gel	The gel does not flow to the bottle cap upon inversion.
**G**	Moderately deformable nonflowing gel	The gel flows about halfway down the bottle upon inversion.
**H**	Slightly deformable nonflowing gel	The gel surface only slightly deforms upon inversion.
**I**	Rigid gel	There is no gel-surface deformation upon inversion.
**J**	Ringing rigid gel	A tuning-fork-like mechanical vibration can be felt after tapping the bottle.

## Data Availability

The original contributions presented in the study are included in the article, further inquiries can be directed to the corresponding authors.
